# Genome-Wide association study of quantitative biomarkers identifies a novel locus for alzheimer’s disease at 12p12.1

**DOI:** 10.1186/s12864-021-08269-8

**Published:** 2022-01-28

**Authors:** Brian Lee, Xiaohui Yao, Li Shen, for the Alzheimer’s Disease Neuroimaging Initiative

**Affiliations:** 1 Department of Biostatistics, Epidemiology and Informatics, Perelman School of Medicine at the University of Pennsylvania, Philadelphia, USA; 2 Alzheimer’s Disease Neuroimaging Initiative Informatics Core, Los Angeles, USA

**Keywords:** Alzheimer’s disease, Genome-wide association study, Quantitative biomarkers, Cognitive traits, Imaging traits

## Abstract

**Background:**

Genetic study of quantitative biomarkers in Alzheimer’s Disease (AD) is a promising method to identify novel genetic factors and relevant endophenotypes, which provides valuable information to deconvolute mechanistic complexity and better understand disease subtypes.

**Results:**

Using the data from the Alzheimer’s Disease Neuroimaging Initiative (ADNI), we performed a genome-wide association study (GWAS) between 565,373 single nucleotide polymorphisms (SNPs) and 16 key AD biomarkers from 1,576 subjects at four visits. We identified a novel locus rs5011804 at 12p12.1 significantly associated with several AD biomarkers, including three cognitive traits (CDRSB, FAQ, ADAS13) and one imaging trait (fusiform volume). Additional mediation and interaction analyses investigated the relationships among this SNP, relevant biomarkers, and clinical diagnosis, confirming and further elaborating the genetic effects seen in the GWAS.

**Conclusion:**

Our GWAS not only affirms key AD genes but also suggests the promising role of the SNP rs5011804 due to its associations with several AD cognitive and imaging outcomes. The SNP rs5011804 has a reported association with adult asthma and slightly affects intracranial volume but has not been associated with AD before. Our novel findings contribute to a more comprehensive view of the molecular mechanism behind AD.

**Supplementary Information:**

The online version contains supplementary material available at (10.1186/s12864-021-08269-8).

## Background

Alzheimer’s disease (AD) is a complex neurodegenerative disease commonly characterized by memory impairments, cognitive decline, and the presence of both tau and A *β* [1]. There is an urgent need for developing effective strategies to discover new AD risk or protective biomarkers for disease modeling and drug development [2]. Genetics plays an important role in AD with estimated heritability in the range of 58–79% [3–5]. Genome wide association studies (GWAS) of case-control status have only discovered about 30 independent genetic factors for AD susceptibility [6–8], which could not explain all the heritability and thus requires scientists to explore alternative search strategies for AD genetic determinants. With the availability of large-scale genetics, imaging, cognition and biomarker data in landmark studies such as the Alzheimer’s Disease Neuroimaging Initiative (ADNI) [9–11], genetic analysis of multidimensional quantitative traits (QT) in AD becomes an emerging and rapidly growing research field [12–14]. The QT approach has distinct advantages in power over categorical diagnoses. For example, genetic studies of AD imaging QTs have yielded some prominent new findings [15–19], including a few contributions to genetically based drug targets [18–21].

Specifically, there are a variety of cognitive, imaging and other biomarkers that can serve as AD-associated QTs, such as Clinical Dementia Rating - Sum of Boxes (CDRSB), Functional Activities Questionnaire (FAQ), and the Alzheimer’s Disease Assessment Scale 13-item Cognitive Subscale (ADAS13) and neuroimaging volumetric measurements (e.g., those of fusiform and entorhinal cortex measured by the FreeSurfer (FS) software) [22]. As many of these measurements have been shown to accurately depict some form of mild or severe cognitive impairment related with dementia [23–26], it is possible to find a specific single nucleotide polymorphism (SNP) highly associated with AD by finding an association between the SNP and one or more indications of cognitive impairments/dementia as determined by these measurements.

Finding associations between SNPs and AD as a whole using specific QTs as a proxy is extremely useful for a couple of reasons. First, these measurable biological properties are often (much) more strongly associated to the pathogenesis of AD as a whole than a single, static diagnosis might be. Additionally, these quantitative continuous variables can account for and depict an individual’s AD status and neurological health more finely than a single diagnosis can, and possibly implicate a disease subtype mechanism. Given the heterogeneity of AD, with only one diagnosis code representing the wide range of mild/intermediate cognitive impairments in addition to the study’s case-control design, this single measure cannot offer the same amount of insight into an individual’s progression status that a QT in the form of a measurable biomarker can, making the many measurements and calculations performed significantly less precise and powerful. Lastly, these QTs are continuous measures and statistically more powerful than case-control status, often requiring much fewer samples for a genetic discovery. This realization is fueled by the assumption that imaging, cognitive and other QTs are closer to the inherent neurobiology of the disease than a diagnosis itself; a previous study [27] has confirmed this assumption in showing there are instances where common genetic variation shows a stronger impact on brain structure than on risk for neuropsychiatric disorders. As such, biologically relevant variants that might not pass stringent multiple-test corrections in the typical case-control studies described here are more likely to be found in an associative study using intermediate biomarkers like this one [28].

Previous studies using data from the ADNI cohort highlighted several key AD genes including APOE, TOMM40, APOC1, BIN1, and CR1 [13] using AD diagnostic data. However, it is possible such studies might have missed some biologically relevant variants. Other studies have replicated these findings using AD neuroimaging data (including but not limited to [29–31]), fluid biomarkers (including [32–34]), or cognitive biomarkers (including [35, 36]). However, not many studies have explicitly measured this larger spectrum of phenotypes across individuals within the same cohort. To bridge this gap, in this work, we perform GWAS analysis on a set of imaging, cognitive and biomarker QTs in ADNI, which are provided by the Quantitative Template for the Progression of AD (QT-PAD) Project (http://www.pi4cs.org/qt-pad-challenge). The QT-PAD includes a set of longitudinal key AD biomarkers for *n*=1,737 ADNI participants. This large amount of normalized biomarker and detailed diagnosis data, when combined with covariates including age, gender and education level, allows researchers to perform significantly more powerful statistical analyses and directly compare GWAS results studying different QTs in AD. Additionally, given the major role the time plays in AD, the ability to study the progression of the disease over time and the corresponding genetic determinants is especially useful.

In summary, although previous GWAS have found various genetic variations that are highly associated with AD, it is possible that certain biologically significant variants that may not have survived the typical case-control study’s corrected *p* value thresholds. As such, in this work, we study a set of key AD QTs including a wide array of cognitive, cerebrospinal fluid (CSF), and imaging biomarkers involved in the QT-PAD project. Using this dataset allows for 1) increased statistical power compared with case-control GWAS studies; 2) the study of a wide variety of leading AD biomarkers, to help de-convolute mechanistic complexity and better understand disease subtypes; and 3) additional longitudinal analyses, to study the progression of the disease and the stability of the genetic determinants over time. Our overarching goal is to not only confirm the known AD genes but also identify novel AD genetic findings.

## Results

### Targeted genetic association

**Fig. 1:**
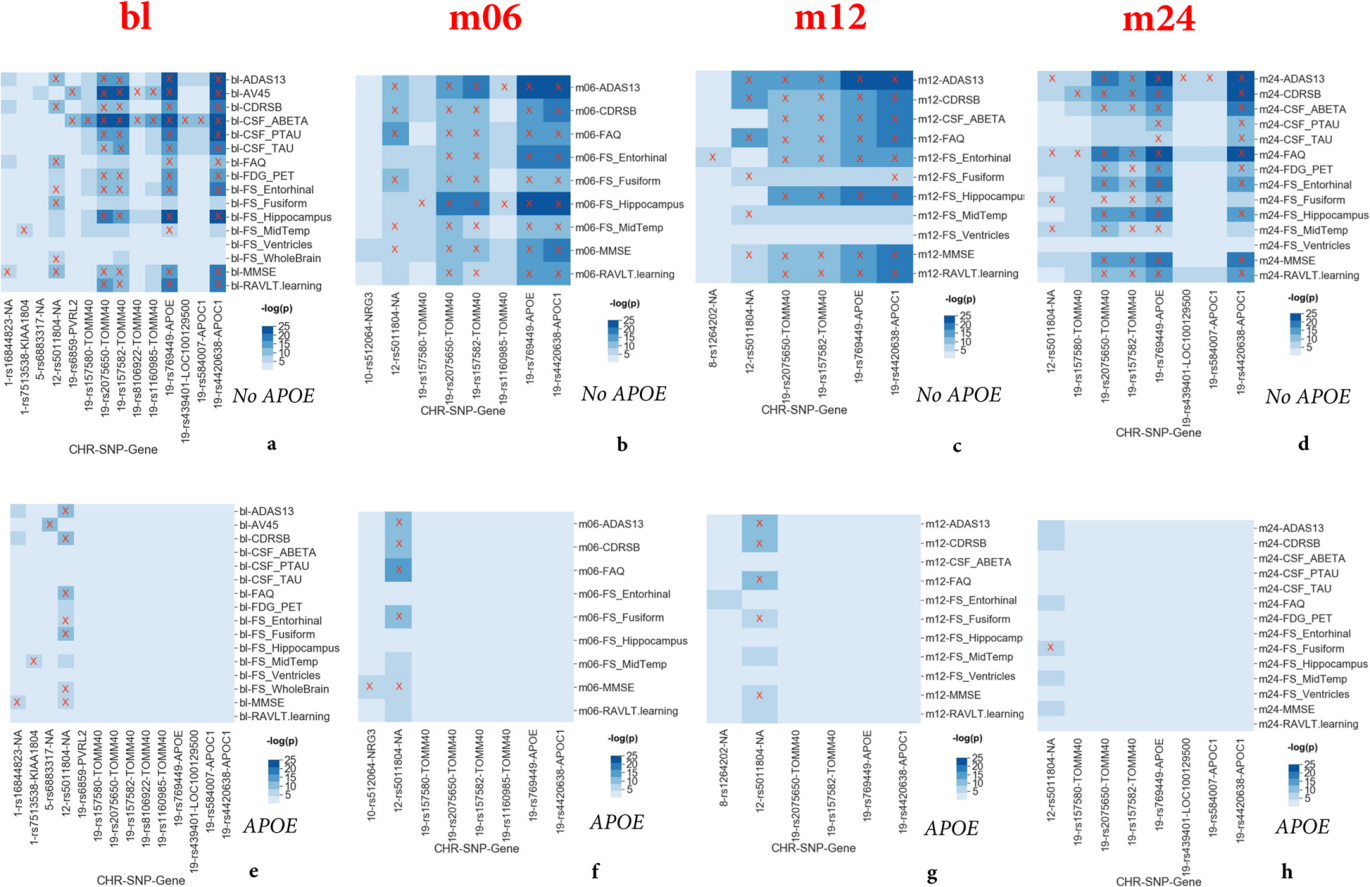
Heatmap showing results of GWAS for bl, m06, m12, and m24 visit data. GWAS results on baseline QT-PAD biomarkers. Entries with *p*<5×10^−8^ (genome-wide significance threshold) are marked with X for each of the four visit codes: bl (**a**), m06 (**b**), m12 (**c**), and m24 (**d**). The results of analyses with three covariates (age, gender, and education) are shown in **a**, **b**, **c**, **d** while the results of analyses with four covariates (age, gender, education, APOE *ε*4) are shown in **e**, **f**, **g**, **h**

GWAS highlighted the effect of rs5011804 at 12p12.1 with several biomarker QTs across all four studied time points. The results of our analyses have been summarized below in two series of heat maps. These figures display all SNP-QT pairings across all four time points (ranging from the baseline visit to two years later) with low *p*-values, where significant pairings (*P*<5×10^−8^, a genome-wide threshold) are marked with a red ‘X’. The first series (Fig. 1a through d) have age, gender, and education as covariates while the second series (Fig. 1e through h) consider age, gender, education, and genetic dosage of APOE *ε*4 as covariates. The analyses that do not control for APOE have 25 statistically significant SNP-outcome pairings across all four time points; the analyses that do control for APOE have 16 statistically significant SNP-outcome pairings across all four time points. *P*-values for the non-APOE analyses are as low as 1.879×10^−14^ (association with CDRSB at the month 12 time point), 2.163×10^−14^ (association with FAQ at the month 12 time point), and 8.211×10^−14^ (association with ADAS13 at the month 12 time point). *P*-values for the analyses that include APOE as a covariates are as low as 1.134×10^−13^ (association with CDRSB at the month 12 time point), 1.262×10^−13^ (association with FAQ at the month 12 time point), and 4.424×10^−13^ (association with ADAS13 at the month 12 time point). One reassuring aspect of our analyses is that in addition to showing the significance of the novel SNP rs5011804 (Fig. 1), we have verified the significance of several variants on chromosome 19 strongly associated with AD, including those from AD genes APOC1, APOE, and PVRL2.

Of note, the SNP rs5011804 remains to be significant in the GWAS that correct for APOE *ε*4 dosage as a covariate; this confirms that the novel SNPs effect is independent from those of APOE *ε*4 allele. All summary statistics from our GWAS can be found in the Supplementary Material.

### Association of rs5011804 with ADAS13, CDRSB, FS Fusiform, and FAQ

**Fig. 2:**
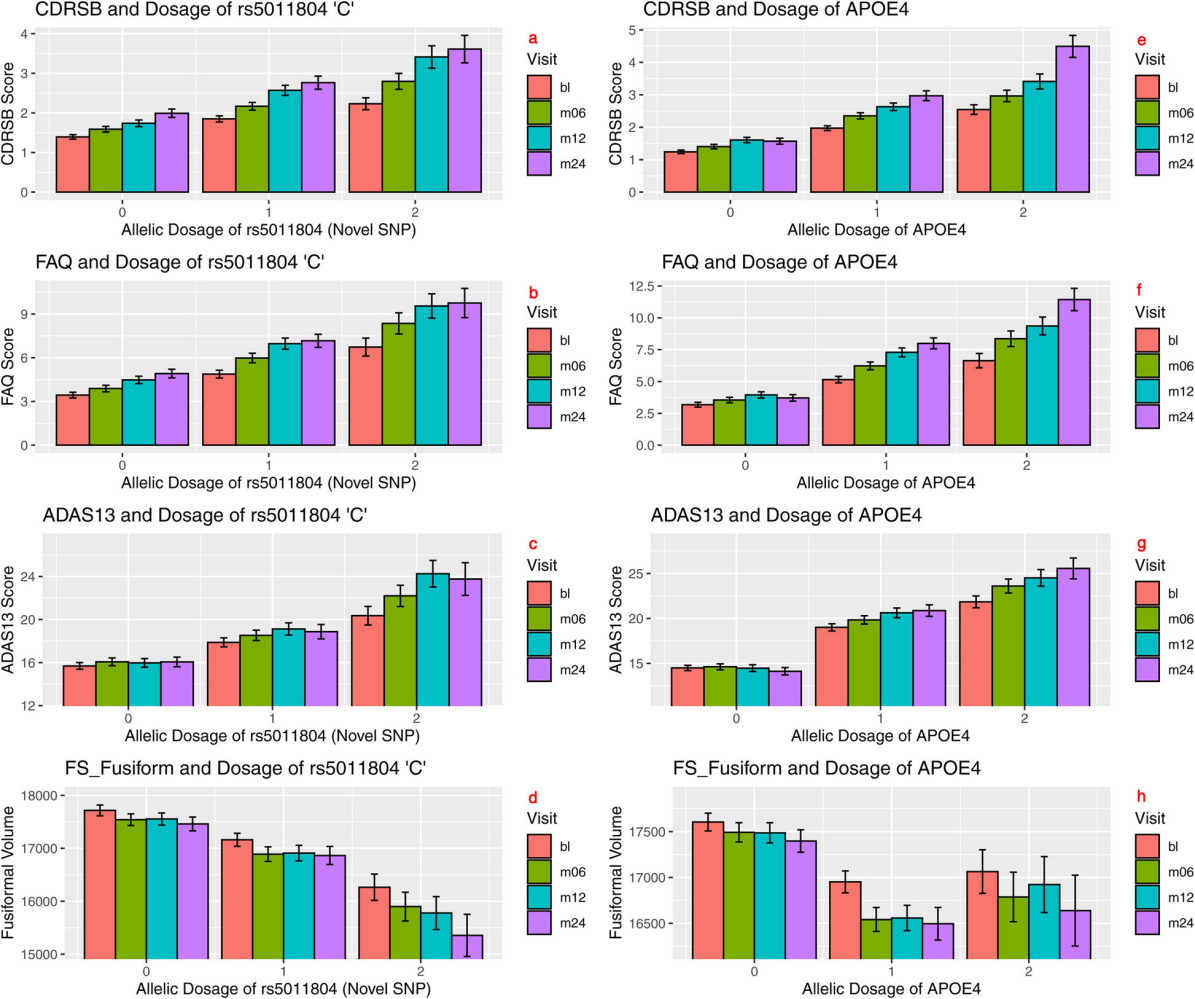
Top QT-PAD biomarkers associated with rs5011804 for all four time points (bl, m06, m12, m24). Mean CDRSB score (**a**), FAQ score (**b**), ADAS13 score (**c**), and FS Fusiform volume (**d**) were plotted against the number of copies for the ‘C’ allele possessed by an individual (**a**-**d**) and the genetic dosage of APOE (**e**-**h**)

To confirm the direction of the effect of the novel locus, we examined a selection of the biomarkers that were strongly associated with the SNP. The biomarkers ADAS13, CDRSB, FS Fusiform, and FAQ had the smallest additive *p*-values out of all measured QTs associated with rs5011804, as evidenced by Fig. 2(a-d), and as such were selected for further analysis.

Individuals with data for a specific biomarker were sorted into one of three categories based on the number of ‘C’ alleles associated with rs5011804 present (0, 1, or 2 copies). The average level of each of four biomarkers was found for each of the three separate categories, which was then plotted along with the standard error of the mean in Fig. 2(a-d). To doubly verify the independence of the novel SNPs effects from APOE *ε*4 dosage, the same procedure was followed except for plotting the genetic dosage of the APOE *ε*4 per individual versus the level of the phenotypic biomarker in Fig. 2(e-h). Additionally, to account for the violation of normality and variance homogeneity in our data, we have plotted a figure similar to Fig. 2 depicting the median and interquartile range instead of the mean and standard error. This updated figure can be seen as Fig. S3 in the Supplementary Material.

To determine the significance of the difference between pairs of averages (i.e. between the average level of a biomarker for individuals with no copies of the novel allele and two copies, individuals with one and two copies, and individuals with no and one copy), Cohen’s d values and two-tailed t-test p statistics were computed (Table 1). Similarly, to determine the significance of the difference between pairs of averages (i.e. between the average level of a biomarker for individuals with no copies of the APOE *ε*4 and two copies, individuals with one and two copies, and individuals with no and one copy), Cohen’s d values and two-tailed t-test p statistics were found (Table 2).

**Table 1:** Mann-Whitney U Test *P* Values for Fig. 2(a-d)

Visit (Dosage)	CDRSB P	FAQ P	ADAS13 P	Fusiform P
bl (0 vs 1)	3.59e-6	1.37e-5	1.08e-4	6.56e-2
bl (0 vs 2)	2.81e-9	1.95e-8	3.01e-8	4.19e-3
bl (1 vs 2)	2.22e-3	2.63e-3	1.29e-3	2.47e-2
m06 (0 vs 1)	8.60e-6	1.23e-6	3.67e-5	2.11e-1
m06 (0 vs 2)	1.58e-11	1.09e-10	2.03e-10	4.39e-4
m06 (1 vs 2)	2.44e-4	5.69e-4	1.56e-4	2.37e-3
m12 (0 vs 1)	1.44e-7	6.34e-7	5.62e-6	1.71e-1
m12 (0 vs 2)	3.79e-10	7.35e-10	1.63e-11	7.55e-5
m12 (1 vs 2)	2.15e-3	2.30e-3	6.14e-5	4.19e-4
m24 (0 vs 1)	5.19e-4	3.26e-4	5.16e-3	2.52e-1
m24 (0 vs 2)	7.50e-8	1.13e-7	3.21e-7	5.12e-4
m24 (1 vs 2)	1.29e-3	1.87e-3	1.03e-3	2.94e-5

The index/leftmost column of the table represents the two specific allelic dosage means being compared at a specific time point. For example, in the first row, the differences in the average baseline levels for each of the four listed phenotypes (CDRSB, FAQ, ADAS13, and FreeSurfer Fusiform) are compared between individuals with 0 copies and 1 copy of the minor allele of the SNP rs5011804. The visit codes have been abbreviated as follows: bl represents baseline measures, m06 represents measures at the month 6 visit, m12 represents measures at the month 12 visit, and m24 represents measures at the month 24 visits. A Mann–Whitney U test *p* value threshold was determined using the Bonferroni correction, setting a threshold of 1.04∗10^−3^

**Table 2:** Mann-Whitney U Test *P* Values for Fig. 2(e-h)

Visit (Dosage)	CDRSB P	FAQ P	ADAS13 P	Fusiform P
bl (0 vs 1)	7.20e-21	6.65e-15	1.26e-22	4.08e-4
bl (0 vs 2)	1.85e-23	4.21e-15	6.82e-25	1.11e-2
bl (1 vs 2)	5.12e-5	2.77e-3	1.22e-4	4.31e-1
m06 (0 vs 1)	1.26e-20	9.61e-16	9.21e-20	2.47e-6
m06 (0 vs 2)	4.70e-25	1.84e-21	4.15e-25	9.68e-4
m06 (1 vs 2)	3.69e-5	3.51e-5	3.23e-5	1.51e-1
m12 (0 vs 1)	5.43e-17	2.81e-17	1.21e-12	8.42e-4
m12 (0 vs 2)	2.97e-20	4.69e-18	7.29e-25	8.60e-2
m12 (1 vs 2)	1.24e-4	9.54e-4	6.87-5	1.41e-1
m24 (0 vs 1)	1.72e-16	3.10e-18	1.05e-20	1.34e-3
m24 (0 vs 2)	9.06e-22	2.10e-19	1.98e-21	6.57e-3
m24 (1 vs 2)	8.18e-6	2.18e-4	6.54e-5	3.85e-2

The index/leftmost column of the table represents the two specific allelic dosage means being compared at a specific time point. For example, in the first row, the differences in the average baseline levels for each of the four listed phenotypes (CDRSB, FAQ, ADAS13, and FreeSurfer Fusiform) are compared between individuals with 0 copies and 1 copy of the minor allele of the APOE *ε*4 allele. A Mann–Whitney U test *p* value threshold was determined using the Bonferroni correction, setting a threshold of 1.04∗10^−3^

The moderately high Cohen’s d values and relatively low two-tailed t test p statistics found, especially between individuals with no and two copies of the relevant minor ‘C’ allele, confirm the significant effect this SNP has. From these visualizations, it is apparent that the rs5011804 ‘C’ allele is associated with higher CDRSB, ADAS13, and FAQ scores, which are indicative of a significant cognitive impairment commonly seen in individuals with AD. The same allele is also associated with significantly lower values of the FS Fusiform biomarker, which is consistent with the atrophy expected in neurodegenerative diseases like AD.

In addition to evaluating the effect this SNP has on biomarkers commonly associated with AD, we examined the relationship between the SNP and the diagnosis at each time point. This was done via a case-control linear regression analysis in PLINK v1.90 [37]. Individuals with diagnoses of mild cognitive impairment (MCI) or dementia (AD) were coded as cases while healthy controls (HC) were coded as the controls. The resulting *p*-value was 1.47×10^−3^, which is significant given a standard Bonferroni-corrected *p*-value threshold of 0.01.

To confirm the significance of these discrete differences, a chi-squared test were performed for the data at each time point. These tests rejected the null hypothesis that the genetic dosage at each time point is independent from the AD diagnosis with *p*<1.00×10^−5^ for the bl, m06, and m12 visit codes and *p*=4.77×10^−4^ for the m24 visit code.

### Mediation analysis

**Fig. 3:**
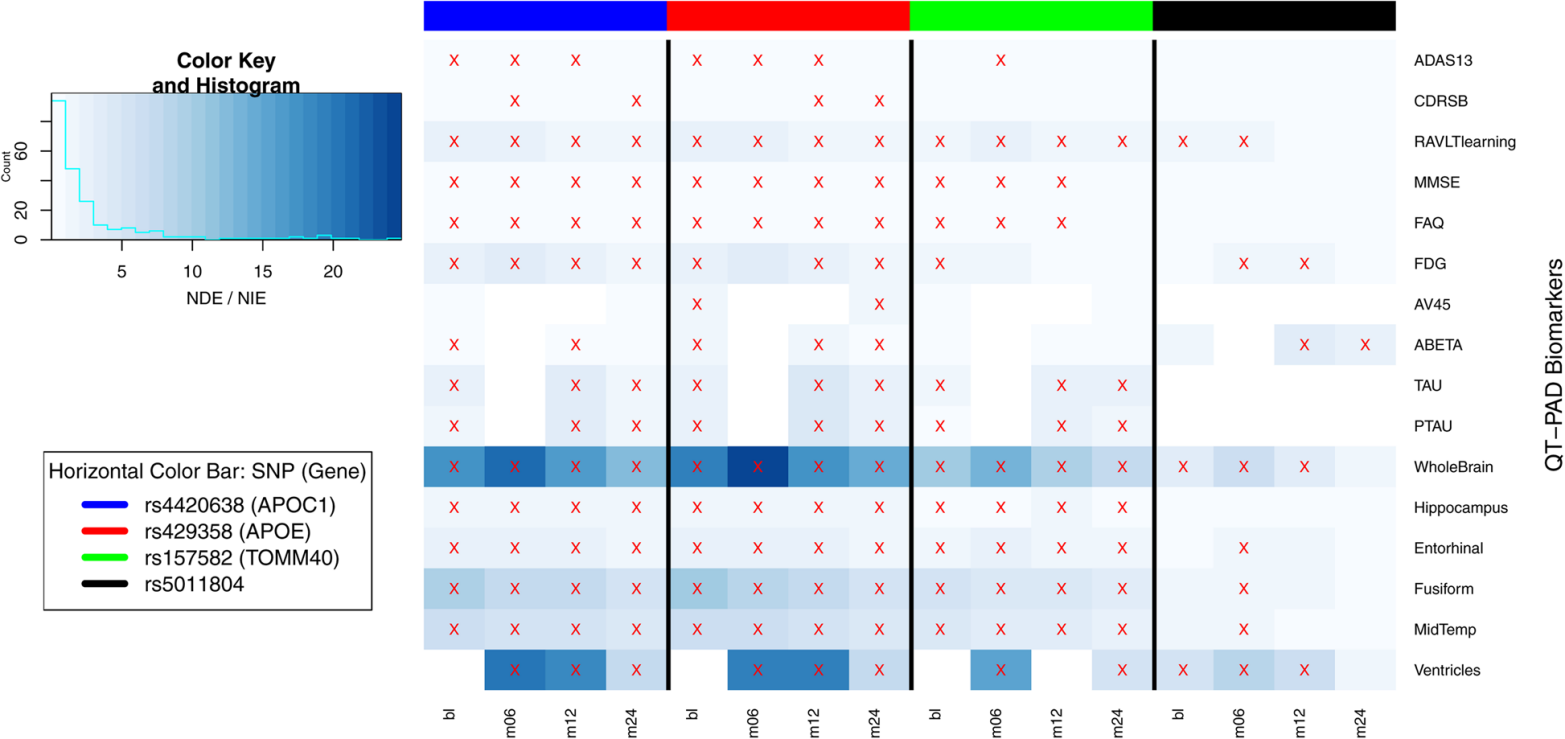
Visualizing the proportion of NDE/NIE versus visit code per QT-PAD outcome. Using the results of the mediation analysis, we plotted the proportion of the natural direct effect (NDE) versus the natural indirect effect (NIE) over time per outcome. Outcomes with a significant NDE and NIE have been marked with a red ‘X‘; the horizontal color bar represents the SNPs for which the mediation effect has been measured. Although the primary findings are focused on rs5011804, the mediation effect coefficients for three other AD SNPs are shown here for reference

Several statistically significant SNP-QT associations (highlighted in Fig. 1(a-d) across all four time points) were found to exhibit a mediation effect. Here the SNP (the independent variable) influences the QT (the mediator variable), which in turn influences the diagnostic outcome (the dependent variable). The proportion of the mediating effect of the QT was calculated and shown in Fig. 3. In this figure, we plot the proportion of the mediation effect calculated against the visit code in question to show the progression of this proportion over time, which both highlights the significance of these effects and confirms the impact of our SNP. We chose to specifically focus on QT outcomes with at least a statistically significant effect at three or more visit codes to ensure significance. Our measurements show that the CDRSB outcome has the largest proportion of the mediating effect. All effects shown and found are consistent with what would be expected in the case of a cognitive dysfunction such as Alzheimer’s.

This specific analysis serves to show that the effect this SNP has on an individual’s AD diagnosis goes through these significant QTs. The discovered QTs mediate the effects of AD candidate variants on disease, which may not be directly detected from the SNP-QT association analysis performed earlier. To the best of our knowledge, this is among the first analysis in AD studies to look for QTs mediating genetic effects on AD diagnosis. We have identified multiple QT mediators linking the SNP to the diagnosis, showing the promise this SNP may have a causal mechanism through these QTs to influence AD diagnosis.

### Interaction analysis

**Fig. 4:**
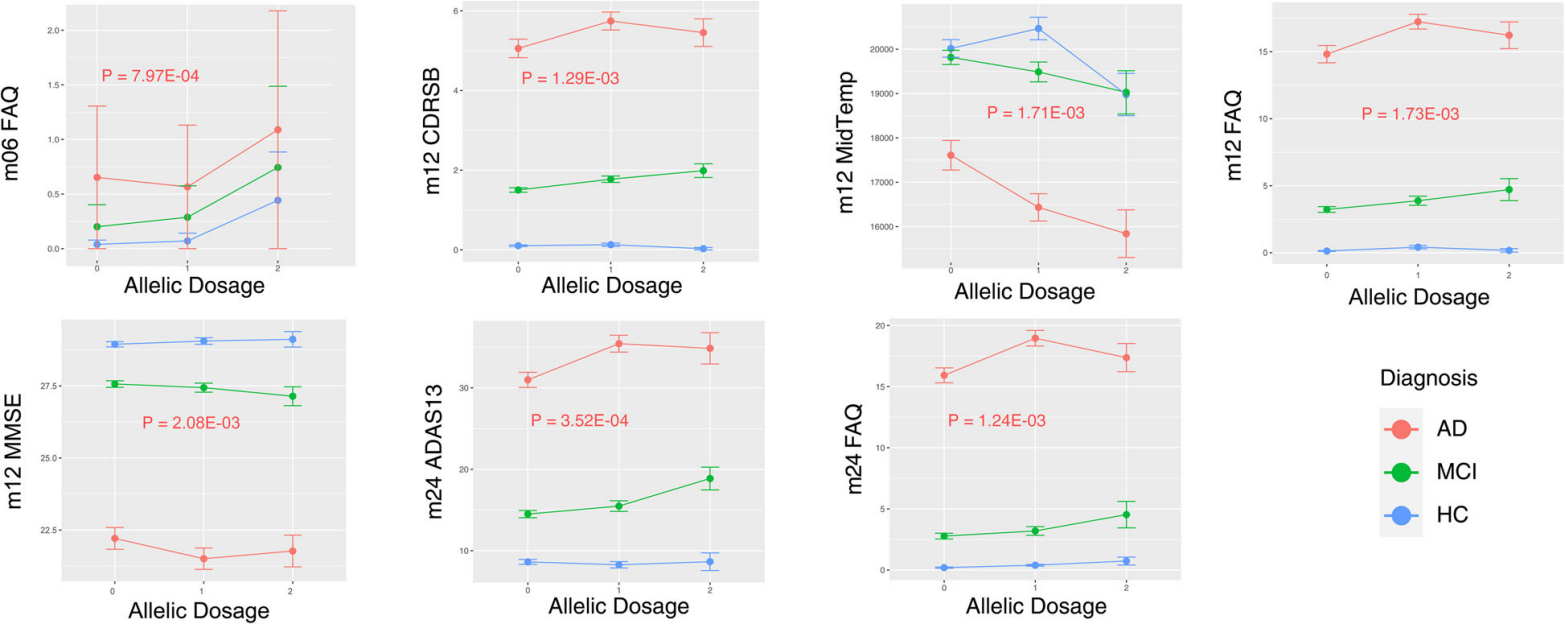
rs5011804-by-diagnosis interaction analysis visualizations. The diagnosis × allelic dosage of the novel SNP rs5011804 was plotted against the average level of biomarker for each of several diagnostic and imaging biomarkers. Adjacent to each graph shown in red is the relevant *p*-value of the interaction effect found. Additionally, a similar figure showing the median and interquartile range has been made and is available in the Supplementary Material (Figure S4)

Our interaction analysis found seven specific SNP-by-diagnosis interaction relationships of statistical significance determined via a Bonferroni correction. Six cognitive and one imaging QTs exhibited such an effect, as shown in Fig. 4. Among these findings, similar interaction patterns are found on all six cognitive QTs, including FAQ measures (at m06, m12 and m24), CDRSB measures (at m12), MMSE measures (at m12) and ADAS13 measures (at m24). Specifically, for these cognitive QTs, while the SNP rs5011804 demonstrates either an additive effect or no effect in both NL and MCI diagnostic groups, it shows a heterozygous effect in the AD group, where heterozygous AD patients (i.e., allelic dosage = 1) have the smallest or largest mean FAQ, compared with homozygous AD patients. In contrast, a different interaction pattern is shown on the only imaging QT finding. For MidTemp measures (at m12), the SNP rs5011804 shows an additive effect in both MCI and AD groups, and shows a heterozygous effect in the NL group.

## Discussion

Our GWAS analysis of targeted AD biomarkers discovered a novel SNP rs5011804 at 12p12.1 associated with measures of several quantiative biomarkers in 1,576 members of the QT-PAD cohort. Our post-hoc stratified, mediation, and interaction analyses have yielded a few observations as follows. First, this effect is independent of common AD risk APOE *ε*4. Second, there is a mediating effect between the SNP and an individual’s AD status through a collection of key biomarkers. Finally there exist strong SNP-by-diagnosis interaction effects on a few AD biomarkers.

A genome-wide association study has implicated that our novel SNP is highly associated with several AD quantitative traits (QTs) with *p*<5.00×10^−8^. To our knowledge, this is the first time this SNP has been reported to be strongly associated with AD.

Previously, this SNP has been strongly associated with adult-onset asthma after correcting for smoking habits [38]. The ENIGMA2 project has previously found that rs5011804 has a borderline association with the intercranial volume (ICV) with a *p*=0.05934 [39]. In their late-onset AD GWAS, Kunkle et al. included rs5011804 in their analyses but did not find that it had a statistically significant effect *p*=0.7015 [6].

To better understand the function of the region of chromosome 12 rs5011804 is in, we attempted to find other SNPs in the same LD block. Using the SNPStats R library [40], we searched for all SNPs within a 1 Mb range that were in linkage disequilibrium with rs5011804, as defined by *D*^′^≥0.8,*r*^2^≥0.8, GWAS *p*≤0.05. No SNPs were found to be in linkage disequilibrium with rs5011804.

Although rs5011804 is not in linkage disequilibrium with any other currently-recognized SNPs, it is located between genes KRAS (distance ≈ 38 KB) and LMNTD1 (distance ≈ 75 KB). KRAS is an oncogene that produces K-Ras, a GTPase associated with the RAS/MAPK pathway, which is instrumental in cell growth and differentiation. As such, KRAS has been shown to be associated with disorders including lung cancers and cholangiocarcinoma [41]. LMNTD1, also commonly referred to as PAS1C1, is a protein-encoding gene involved in cell population proliferation that is also associated with lung cancer [42].

To gain more insight into the potential regulatory role of rs5011804, data from the Genotype-Tissue Expression (GTEx) Project was studied. The data analyzed was sourced from the GTEx Portal on 11 November 2020. There were no significant variant-gene associations found in any of the provided brain tissues.

In addition to examining the novel SNP, it is essential to discuss the multiple QTs involved in this study. Multiple QTs associated with the SNP and included in the original QT-PAD dataset are strongly linked with AD, and as such, it both makes sense and is expected that a SNP highly associated with some of these QTs would also have a strong association with AD directly. For example, the biomarkers ADAS13, FAQ, CDRSB, MMSE, and RAVLT.learning are diagnostic scales commonly used by physicians to quickly assess a patient’s mental status and can accurately differentiate between healthy individuals from those with mild cognitive impairments or severe dementia [23–25, 43, 44]. The biomarkers FDG PET, Amyloid PET, FS WholeBrain, FS Hippocampus, FS Entorhinal, FS Ventricles, FS MidTemp, and FS Fusiform are either molecular imaging measurements or neuroimaging volumetric measurements; previous studies have shown that specific neurological abnormalities can be an effective and accurate way to diagnose a patient with AD [26, 45, 46]. The final three remaining biomarkers included in QT-PAD – CSF ABETA, CSF TAU, and CSF PTAU – are measurements of A *β*42 and other proteins that are among the best indicators for AD [22]. Given their high association with AD and quantitative nature, these biomarkers are excellent outcomes variables to use in GWAS.

With the advent of Big Data, genome-wide association studies have quickly became the most commonly accepted method to analyze genetic data in order to learn about the genetic etiology of complex diseases. As helpful and implicating as GWAS may be, however, it is necessary to remember that these studies are purely associative.

As such, it is usually necessary to employ additional procedures to confirm and further elaborate the genetic signal(s) seen in the GWAS.

The use of two post hoc analyses in the case of this study attempts to do so. Specifically, on one hand, we performed mediation analysis and discovered multiple imaging and cognitive traits that mediate the SNP effect on the diagnosis, showing the promise the SNP rs5011804 may have a causal mechanism through these traits to influence AD diagnosis. On the other hand, we performed a subsequent SNP-by-diagnosis interaction analysis on the studied biomarkers, and revealed several differential SNP-biomarker association patterns in different diagnostic groups. These findings have great potential to help deconvolute mechanistic complexity and better understand disease subtypes.

This study does not claim to prove there is a definite, unambiguous causal relationship between the novel SNP rs5011804 and an AD diagnosis. Determining all of the multiple biological and environmental factors of an AD diagnosis would require several more studies. Although significant in our cohort, it is necessary for this SNP and the QT’s studied to be examined in the context of other non-Caucasian and European ethnic groups. Further replication studies can confirm if this SNP, along with some of the implicated neuroimaging biomarkers, could help hint at a potential AD mechanism-of-action worth studying. As the SNP both has a significant effect on AD diagnosis through several AD outcomes measured (seen in the mediation analysis) and exhibits a significant effect on AD outcomes when combined with an individual’s diagnosis (as demonstrated in the interaction analysis), perhaps the SNP may assist in determining an individual’s risk for AD in the future.

## Conclusions

In conclusion, we discovered a novel locus rs5011804 at 12p12.1 significantly associated with levels of CDRSB, FAQ, FS Fusiform, and ADAS13 at multiple studied time points including the baseline, month 06, month 12, and month 24 visits in the ADNI cohort. This locus was also found to be strongly associated with an AD diagnosis. Post hoc mediation and interaction analyses confirmed and elaborated the results of our GWAS. In particular, the genetic effect of this SNP on the AD phenotype is mediated by multiple quantitative biomarkers, suggesting possible causal mechanisms from the SNP to biomarkers and to the diagnostic outcome. In addition, differential SNP-biomarker association patterns are identified in different diagnostic groups, providing valuable information for mechanistic understanding of the disease and heterogeneity. This SNP has never been associated with AD before, and our findings may help lead to a more comprehensive view of the molecular mechanism behind AD.

## Methods

**Fig. 5:**
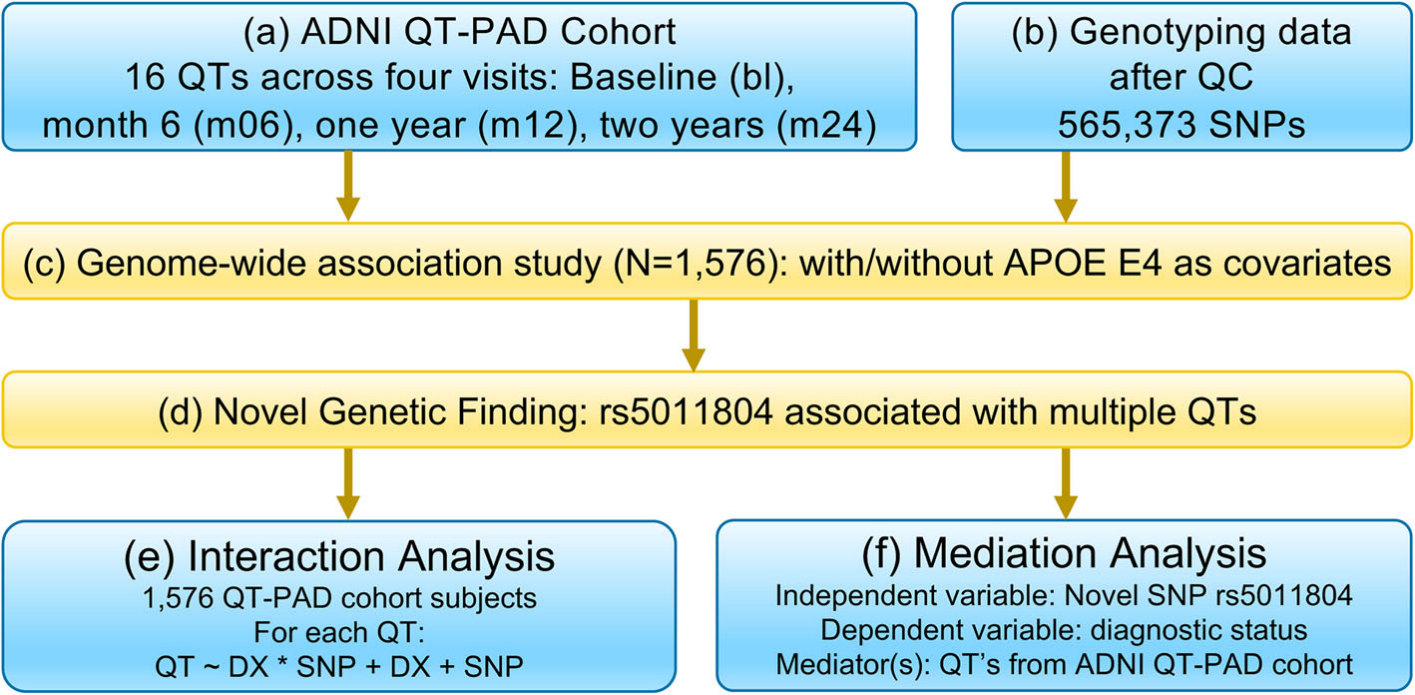
Workflow. A schematic workflow of the analyses performed in this study

All methods were performed in accordance with the relevant guidelines and regulations. Data were downloaded and analyzed under approval of the University of Pennsylvania Institutional Review Board. An overview of the procedure for this study is shown in Fig. 5. Briefly, after identifying four specific time points to examine data for, multiple GWAS were performed using 1,576 individuals from the QT-PAD cohort and using 16 QTs included in QT-PAD. We performed two sets of these GWAS: one set included APOE *ε*4, age, gender, and education as covariates; and the other included just age, gender, and education as covariates. After these GWAS, we also performed mediation analysis between the SNP and diagnosis using a QT-PAD biomarker as the mediator variable, and interaction analysis measuring the SNP-by-diagnosis interaction effect on the QT-PAD biomarkers.

### Alzheimer’s disease neuroimaging initiative QT-PAD data

Data used in this analysis was obtained from the Alzheimer’s Disease Neuroimaging Initiative (ADNI) database [47]. ADNI was launched in 2003 as a public-private partnership led by Principal Investigator Michael W. Weiner, MD to test whether serial MRI, PET, and biological markers can be combined with clinical and neuropsychological assessments to accurately measure the progression of mild cognitive impairment (MCI) and early AD. For up-to-date information, see http://www.adni-info.org.

Participants included individuals who were members of ADNI 1/GO/2 cohorts, as described by the ADNI QT-PAD project (Fig. 5 Box (a)). Please refer to [48] for details about the QT-PAD data and how participants were chosen. Table 3 shows the 16 AD outcomes included in the QT-PAD. To reduce the likelihood of population stratification effects, only non-Hispanic Caucasian participants were involved in this study. As such, there were 1,576 individuals who were studied in each of the four time points. 461 of these individuals are healthy controls (HC) and the remaining 1,115 individuals had either an MCI or AD diagnosis. Demographic data about the individuals included in our analyses can be found in Table 4.

**Table 3:** Description of QT-PAD outcomes/biomarkers, their abbreviations, and categories

Abbreviation	Outcome/Biomarker Description	Category
ADAS13	Alzheimer’s Disease Assessment Scale - Cog 13	Cognition
CDRSB	Clinical Dementia Rating - Sum of Boxes	Cognition
RAVLT.learning	Rey Auditory Verbal Learning Test	Cognition
MMSE	Mini-Mental State Examination/Folstein Test	Cognition
FAQ	Functional Activities Questionnaire	Cognition
FDG PET	Fluorodeoxyglucose Positron Emission Tomography	PET
Amyloid PET/AV45	Amyloid Positron Emission Tomography	PET
CSF ABETA	Cerebrospinal Fluid Beta-Amyloid 42	CSF
CSF TAU	Cerebrospinal Fluid Tau	CSF
CSF PTAU	Cerebrospinal Fluid Phosphorylated Tau	CSF
FS WholeBrain	FreeSurfer Whole Brain Volume	MRI
FS Entorhinal	FreeSurfer Entorhinal Cortex Volume	MRI
FS Ventricles	FreeSurfer Ventricular Volume	MRI
FS MidTemp	FreeSurfer Middle Temporal Gyrus Volume	MRI
FS Fusiform	FreeSurfer Fusiform Volume	MRI
FS Hippocampus	FreeSurfer Hippocampus Volume	MRI

**Table 4:** ADNI QT-PAD Participant Characteristics. Gender, age (in years), education (in years), and genetic dosage of the APOE *ε*4 allele at the baseline are shown

Diagnosis	HC	MCI	AD	N/A
Number	461	797	312	6
Gender (% Female)	49.67	40.15	42.95	black66.67
Age (Mean ±std)	74.47 ±5.68	73.09 ±7.54	75.18 ±7.79	72.15 ±8.29
Education (Mean ±std)	16.42 ±2.65	16.00 ±2.82	15.24 ±2.96	16.75 ±1.50
APOE *ε*4 Dosage (Mean ±std)	0.31 ±0.51	0.62 ±0.68	0.85 ±0.71	0.25 ±0.50

Individuals have been sorted into strata depending on their diagnosis at the baseline visit: healthy control (HC), mild cognitive impairment (MCI), Alzheimer’s Disease (AD), or not applicable for individuals who do not have baseline diagnosis data enclosed (N/A). The mean and standard deviation (std) are provided for the quantitative measures

Genotyping data (Fig. 5 Box (b)) were quality-controlled, imputed using 1000G data, and combined as described in [49, 50]. Briefly, genotyping was performed on all ADNI participants following the manufacturer’s protocol using blood genomic DNA samples and Illumina GWAS arrays (610-Quad, OmniExpress, or HumanOmni2.5-4v1) [51]. Quality control was performed in PLINK v1.90 [37] using the following criteria: 1) call rate per marker ≥95*%*, 2) minor allele frequency (MAF) ≥5*%*, 3) Hardy Weinberg Equilibrium (HWE) test P ≤1.0E-6, and 4) call rate per participant ≥95*%*. In this study, we analyzed the genetic markers available on the ADNI-1 610-Quad panel, where a total of 565,373 SNPs were included in the GWAS.

### Genome wide association studies

To analyze data from multiple time points, multiple GWAS (Fig. 5 Box (c)) were performed, with one analysis per each of the four time points bl, m06, m12, and m24, which represented the baseline, month 6, month 12, and month 24 visits. Due to the large extent of ADNI as a whole and the difficulties each individual patient might have had, not every patient has a recorded value in QT-PAD stored for each of the 16 biomarkers at each visit. To ensure our analyses to have enough statistical power, we only studied time points with a minimum of 200 individuals. As such, our analysis was limited to the four aforementioned time points.

Targeted genetic association analysis of each of the 16 AD biomarkers at each of the four listed time points on the 565,373 SNPs was tested using linear regression under an additive genetic model in PLINK v1.90 [37]. Initially, age, gender, and education only were used as covariates in our GWAS. To correct for the effects of APOE *ε*4 status (best known AD genetic risk factor), GWAS was also performed using the exact same data with age, gender, education, and APOE *ε*4 dosage as covariates. For both trials, significant SNP-QT associations were reported using the genome-wide significance threshold of *p*≤5.00×10^−8^.

### Mediation analysis

Given the dynamic nature of Alzheimer’s disease, a cohort may have a varying distribution along the HC-MCI-AD spectrum as seen through varying biomarker levels and changing diagnoses. In the context of a GWAS, having a dynamic phenotype but a static genetic basis over time can seem contradictory, allowing for the possibility of certain genetic factors being significantly associated with an AD diagnosis or biomarkers closely linked to AD at one time point but not another. In order to accommodate such an effect and verify the highlighted SNP indeed plays a role in AD diagnosis and outcomes at all significant time points, we propose a mediation analysis.

**Fig. 6:**
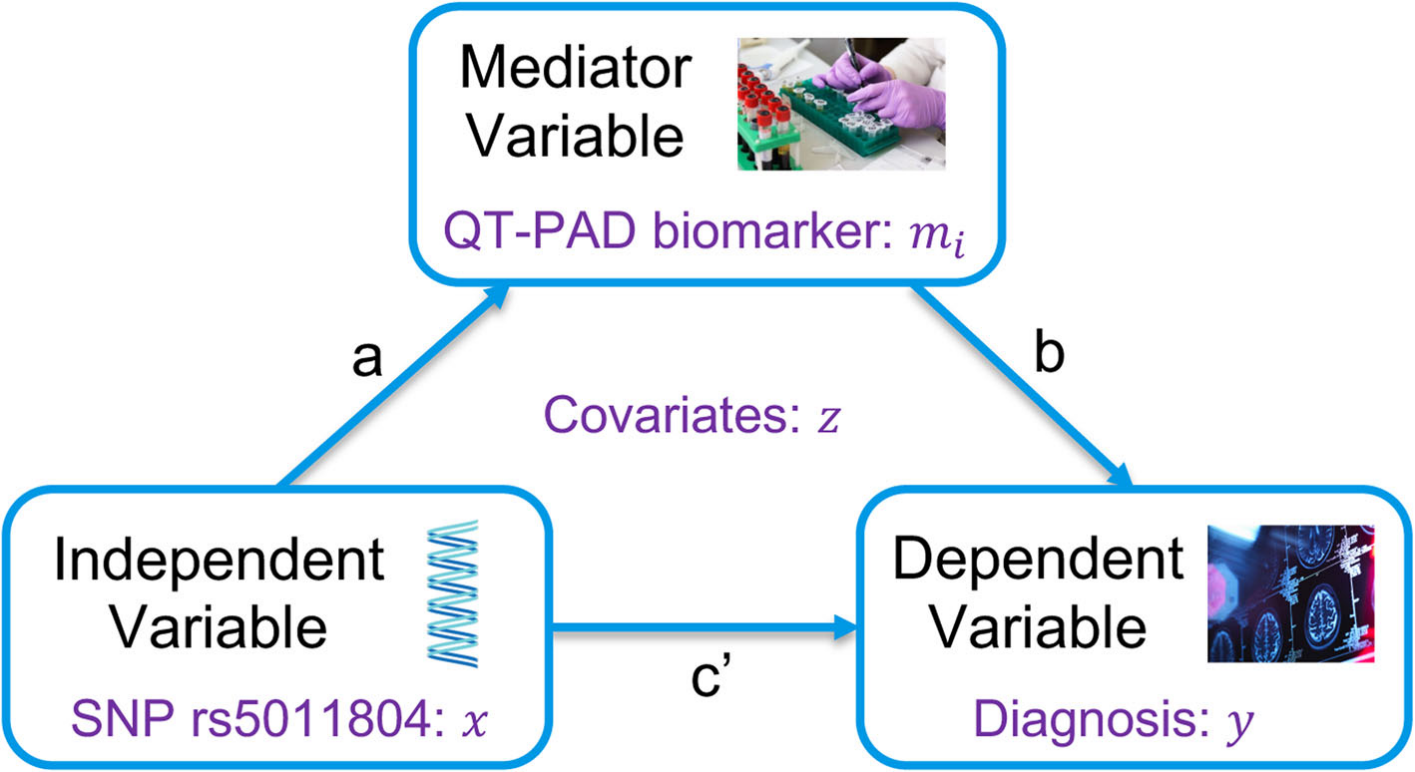
Mediation analysis. Our mediation analysis aims to determine if an independent variable (SNP rs5011804) affects a dependent variable (diagnosis) ‘through’ a mediator variable (QT-PAD biomarker), where age, gender and education are included as covariates. The direct effect is the path coefficient *c*^′^. The indirect effect is the path coefficent product *a*×*b*

A mediation analysis seeks to identify and explain the mechanism of the quantified relationship between rs5011804 and an AD diagnosis via examining the mediating effects of the various ADNI QT-PAD biomarkers discussed (see Fig. 6). Specifically, a mediation analysis will allow us to determine if an independent variable (the SNP rs5011804) affects a dependent variable (an individual’s AD diagnosis) ‘through‘ one of mediator variables (our biologically-motivated AD outcomes). Since many of these QT-PAD outcomes and biomarkers have distinct biological ties to the disease itself, a mediation analysis would both hint at a causal relationship between SNP and diagnosis as well as hint at a possible mechanism of action, drug target, or region of interest (ROI). Below we summarize the specific mediation analysis performed (Fig. 5 Box (f)). For each time point, we followed [52] to perform a standard mediation analysis to identify key biomarkers included in QT-PAD as potential disease moderators. Figure 6 shows a brief graphical summary of this method.

Let *y*∈{1,2} be the dependent variable which represents the diagnostic phenotype in the study, with 2 representing a case (diagnosis of either MCI or AD) and 1 representing a healthy control; *x*∈{0,1,2} be the independent variable which represents the allelic dosage of the minor allele ‘C’ in our identified SNP rs5011804, with 2 signifying an individual has two copies of the minor allele, 1 signifying an individual has one copy of the SNP, and 0 signifying the individual does not carry the variation; z be the covariates (age, gender, and education but not APOE *ε*4 dosage); and *M* be the set of significant biomarkers at each of the four time points as indicated by the previous GWAS. Mediation analysis was performed via the three steps below:

**Step 1:** We use a logistic regression model to regress an individual’s diagnosis *y* against the SNP *x* while controlling for the covariates *z*.

1 $$ logit\left(Pr\left(y=2\right)\right)=\beta_{11}x+\beta_{12}z+\epsilon_{1}  $$

Coefficient *β*_11_ should be significant (*p*-value <0.05) to pass this first step.

**Step 2:** We use a linear regression model to regress each of the potentially mediating biomarkers denoted *m*_*i*_ (i.e., *m*_*i*_∈*M*) against the SNP *x* while controlling for the covariates *z*.

2 $$ m_{i}=\beta_{21,i}x+\beta_{22,i}z+\epsilon_{2,i}  $$

We only use the SNP rs5011804 – and therefore continue with this post hoc analysis – if it meets the significance threshold of 0.05. Coefficient *β*_21,*i*_ should be significant after correcting for the multiple biomarkers; we correct our *p*-value threshold using a Bonferroni correction. As such, given that the number of biomarkers at each step differ, we have a threshold of $\frac {0.05}{7}=7.14\times {10}^{-3}$ for the baseline data, a threshold of $\frac {0.05}{6}=8.33\times {10}^{-3}$ for the m06 and m12 data, and a threshold of $\frac {0.05}{4}=1.25\times {10}^{-2}$ for the m24 data.

**Step 3:** We use a logistic regression model to regress an individual’s diagnosis *y* against the SNP *x* and each mediating biomarker phenotype *m*_*i*_, controlling for the covariates *z*.

3 $$ logit\left(Pr\left(y=2\right)\right)=\beta_{31,i}x+\beta_{32,i}m_{i}+\beta_{33,i}z+\epsilon_{3,i}  $$

Note that this step is only performed using all mediating phenotypes that satisfy the conditions of the previous step. To adjust for multiple comparisons, we again employ the Bonferroni correction to our significance threshold using the number of mediators surviving the previous step. For any given mediating biomarker *m*_*i*_, there is likely a mediating relationship if:

- *β*_32,*i*_ is statistically significant as deemed by our Bonferroni-corrected threshold
- |*β*_31,*i*_|<|*β*_11_| (from Step 1, above). In other words, an indirect effect must be present between our dependent variable and our independent variable through our mediator.

Next, it is possible to compare the multiple mediation effects we have isolated as introduced in [53]; see also Fig. 6. We have also calculated the proportion of the Natural Direct Effect (NDE), which is expressed as *β*_31,*i*_, to the Natural Indirect Effect (NIE) *β*_32,*i*_×*β*_21,*i*_.

This analysis was performed for all significant SNP-QT associations from our GWAS.

### Interaction analysis

Lastly, in addition to examining the main effect of the SNP on the 16 QT-PAD outcomes, we also perform a SNP-by-diagnosis analysis on these QTs (denoted *L*_*QT*_). We primarily consider the novel SNP rs5011804, modeling its allelic effect *x*_*a*_ by coding the genotypes as *x*_*a*_=0,1,2. Similarly, we code an individual’s diagnosis as *x*_*d*_=0,1,2, with HC individuals being coded as 0, individuals diagnosed with MCI as 1, and individuals with an AD diagnosis being coded as 2. By multiplication, we obtain the interaction term *x*_*a*_*x*_*d*_ which represents interaction between the allelic effect of the SNP and an individual’s diagnosis.

As such, we employ the following model to measure the interaction effect *x*_*a*_*x*_*d*_ while also controlling for age (*c*_*age*_), gender (*c*_*gen*_), and education (*c*_*edu*_).

4 $$ L_{QT} = \beta_{0} + \beta_{1} x_{a} + \beta_{2} x_{d} + \beta_{int} x_{a} x_{d} + c_{age} + c_{gen} + c_{edu}  $$

We wish to determine the significance of *β*_*int*_. Given that we are building multiple models, we use a Bonferroni correction to filter out false positives. As we examine QTs from each time point separately, the Bonferroni threshold is calculated as 0.05 divided by the number of statistically significant QTs in a specific visit as determined by the calculations above. Inspiration for this model was taken from [54].

## Supplementary Information

**Additional file 1** Supplementary Material.

## Data Availability

Data used in the preparation of this article were downloaded from the Alzheimer’s Disease Neuroimaging Initiative (ADNI) database. The ADNI data are available to the public at http://adni.loni.usc.edu/ through completion of an online application form and acceptance of Data Use Agreement.

## Abbreviations

AD: Alzheimer’s Disease
ADNI: Alzheimer’s Disease Neuroimaging Initiative
GWAS: genome-wide association study
SNPs: single nucleotide polymorphisms
QT: quantitative trait(s)
CDRSB: Clinical Dementia Rating - Sum of Boxes
FAQ: Functional Activities Questionnaire
ADAS13: Alzheimer’s Disease Assessment Scale 13-item Cognitive Subscale
FS: FreeSurfer (software)
QT-PAD: Quantitative Template for the Progression of Alzheimer’s Disease
CSF: cerebrospinal fluid
HC: healthy control
MCI: mild cognitive impairment
bl: baseline visit
m06: month 6 visit
m12: month 12 visit
m24: month 24 visit
ENIGMA2: Enhancing Neuro Imaging Genetics Through Meta Analysis (Second Continuation)
ICV: intercranial volume
LD: linkage disequilibrium
Mb: megabase(s)
GTEx: Genotype-Tissue Expression (Project)
