# Supplementary material for: Genome-Wide association study of quantitative biomarkers identifies a novel locus for alzheimer’s disease at 12p12.1
Source: BMC Genomics. 2022 Jan 28;23:85. doi: 10.1186/s12864-021-08269-8 (PMC8796646; doi:10.1186/s12864-021-08269-8)
Supplement: Supplementary file 1 — Additional file 1 Supplementary Material. [file 12864_2021_8269_MOESM1_ESM.pdf]

Genome-Wide Association Study of Quantitative Biomarkers Identifies a Novel Locus for Alzheimer's Disease at 12p12.1

Brian Lee[a], Xiaohui Yao[a], Li Shen[a,*], for the Alzheimer's Disease Neuroimaging Initiative[1]

[a] Department of Biostatistics, Epidemiology and Informatics, Perelman School of Medicine, University of Pennsylvania, Philadelphia, PA 19104, USA
[1] Data used in preparation of this article were obtained from the Alzheimer's Disease Neuroimaging Initiative (ADNI) database (adni.loni.usc.edu). As such, the investigators within the ADNI contributed to the design and implementation of ADNI and/or provided data but did not participate in analysis or writing of this report. A complete listing of ADNI investigators can be found at: http://adni.loni.usc.edu/wp-content/uploads/how_to_apply/ADNI_Acknowledgement_List.pdf

[*] Correspondence to:
Li Shen, B306 Richards Building, 3700 Hamilton Walk, Philadelphia, PA 19104, USA;
E-mail: Li.Shen@pennmedicine.upenn.edu

Supplementary Data: Genome-wide association study summary statistics

Please find the results of our genome-wide association study in the attached Excel spreadsheet. These are the results of a PLINKv1.9 GWAS via the --linear tag; details about the format of these output files can be found at [1]. This Excel spreadsheet is organized into eight sheets based on the visit code the data is from (one of ‘bl’, ‘m06’, ‘m12’, ‘m24) and set of covariates used (i.e. those that include APOE4 as a covariate and those that don’t). More information can be found in the sheet titled ‘INFO.’

Supplementary Visualizations – Interaction analysis figures and data

Figure S1: Below we have included visualizations of all (13) identified interaction effects between DX*SNP (*Supplementary Figures a through m)* using the procedure identified.

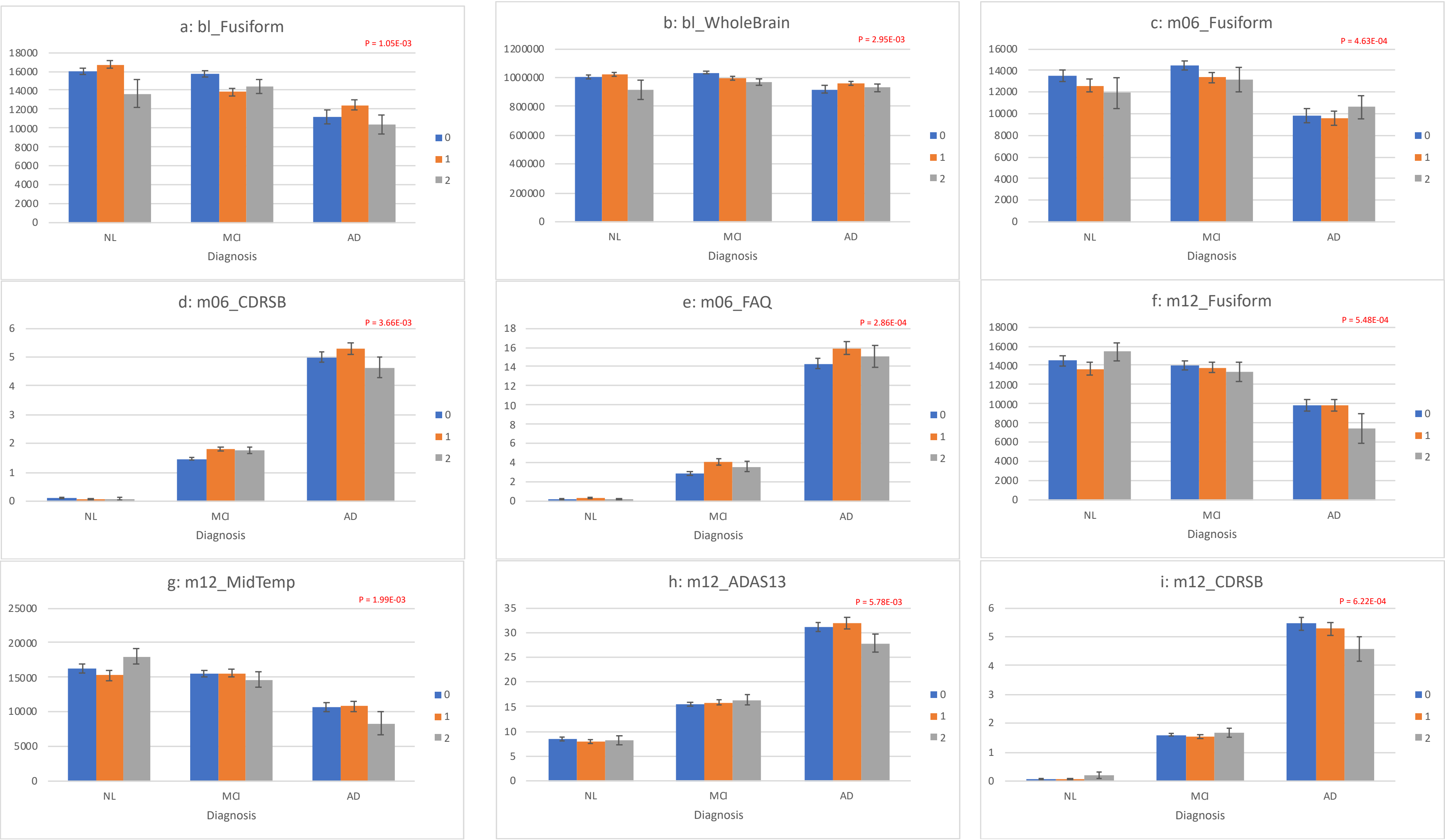

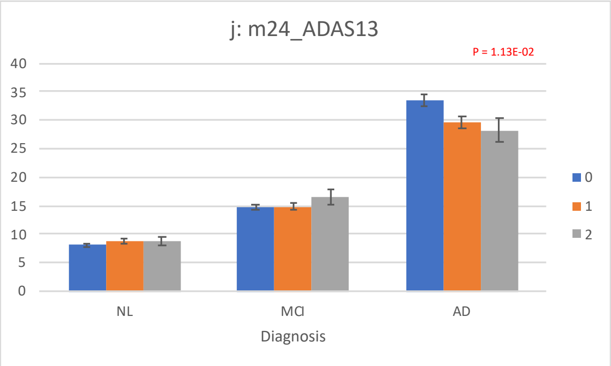
j: m24_ADAS13
P = 1.13E-02
40
35
30
25
20
15
10
5
0
NL
MCI
AD
Diagnosis
0
1
2

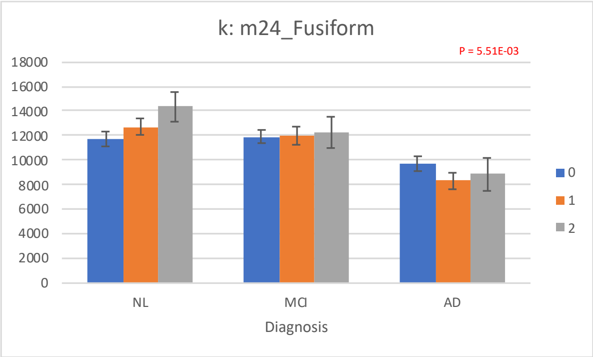
k: m24_Fusiform
P = 5.51E-03
18000
16000
14000
12000
10000
8000
6000
4000
2000
0
NL
MCI
AD
Diagnosis
0
1
2

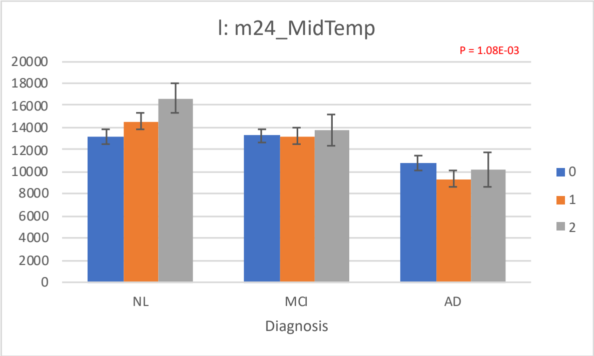
l: m24_MidTemp
P = 1.08E-03
20000
18000
16000
14000
12000
10000
8000
6000
4000
2000
0
NL
MCI
AD
Diagnosis
0
1
2

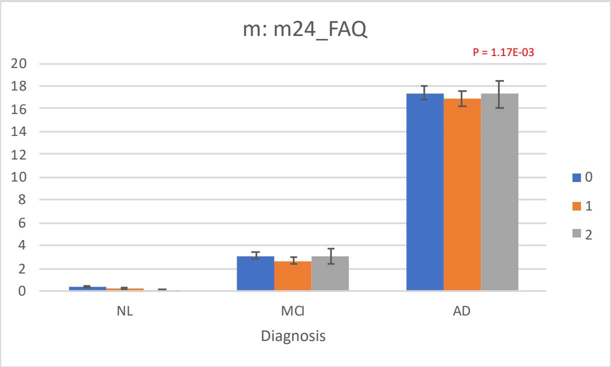
m: m24_FAQ
P = 1.17E-03
20
18
16
14
12
10
8
6
4
2
0
NL
MCI
AD
Diagnosis
0
1
2

Supplementary Visualizations – Manhattan and Quartile-Quartile Plots of Key QT-PAD Biomarker Associations

Figure S2: Below we include Manhattan plots and Quartile-Quartile (Q-Q) plots for key QT-PAD biomarker associations. The significance of novel SNP rs5011804 is present in the Manhattan plots of multiple biomarkers and the Q-Q plots show the results are not significantly inflated.

Baseline ADAS13:

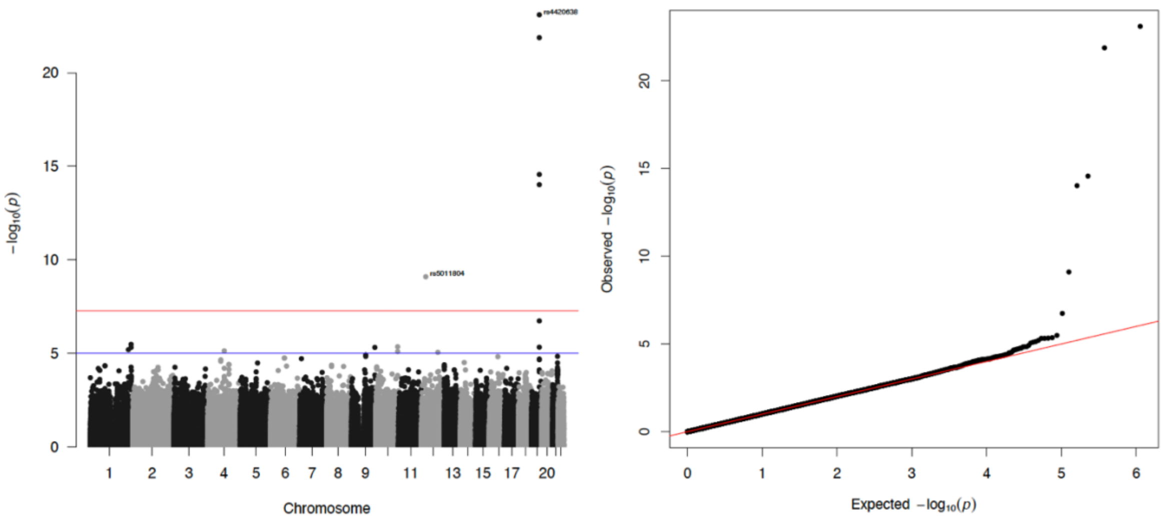

Baseline CDRSB:

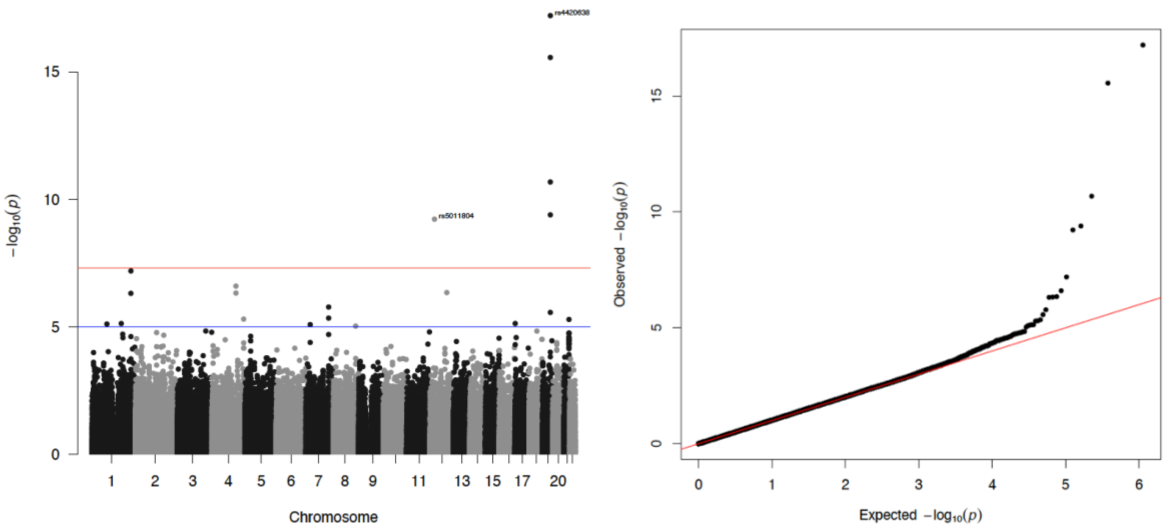

Month 6 Fusiform:

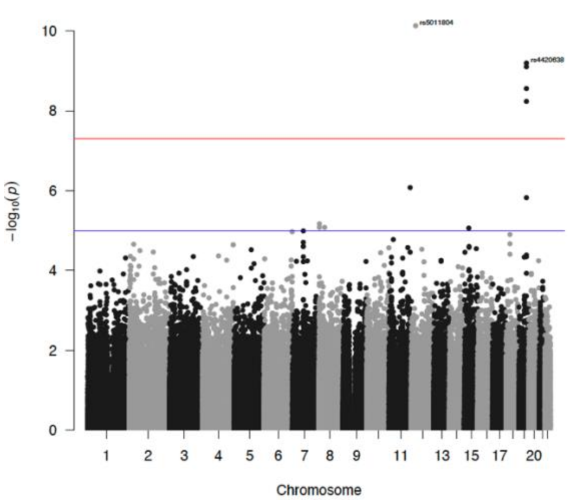

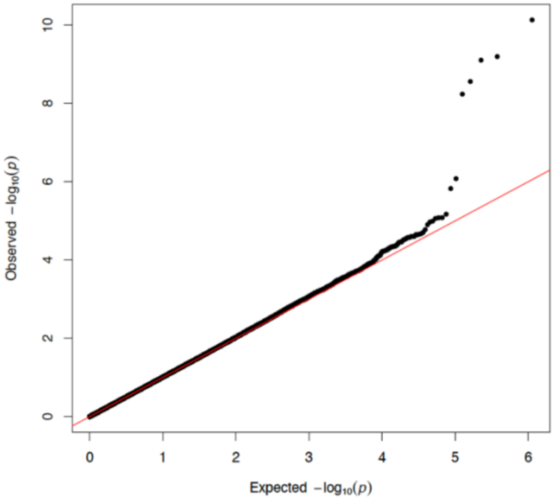

Month 12 FAQ:

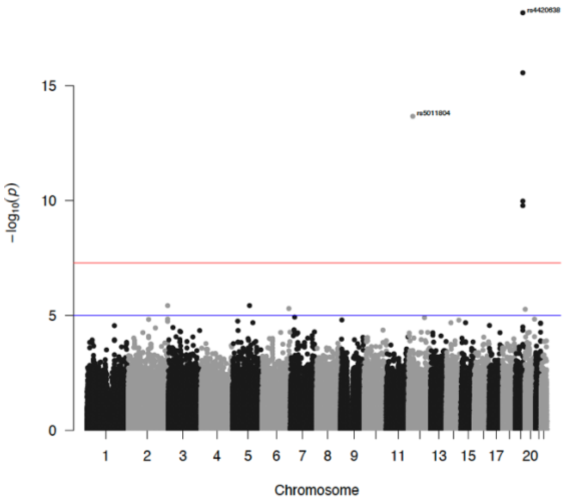

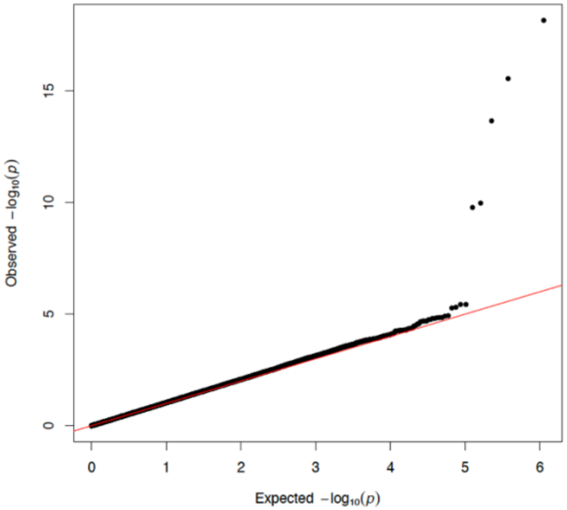

Supplementary Visualizations – Median and Interquartile Range (IQR) Versions of Figures 2 and 4

Figure S3: Top QT-PAD biomarkers associated with rs5011804 for all four time points (bl, m06, m12, m24). Median CDRSB score (a), FAQ score (b), ADAS13 score (c), and FS Fusiform volume (d) were plotted against the number of copies for the ‘C’ allele possessed by an individual (a − d) and the genetic dosage of APOE (e − h). The blue error bars report the interquartile range.

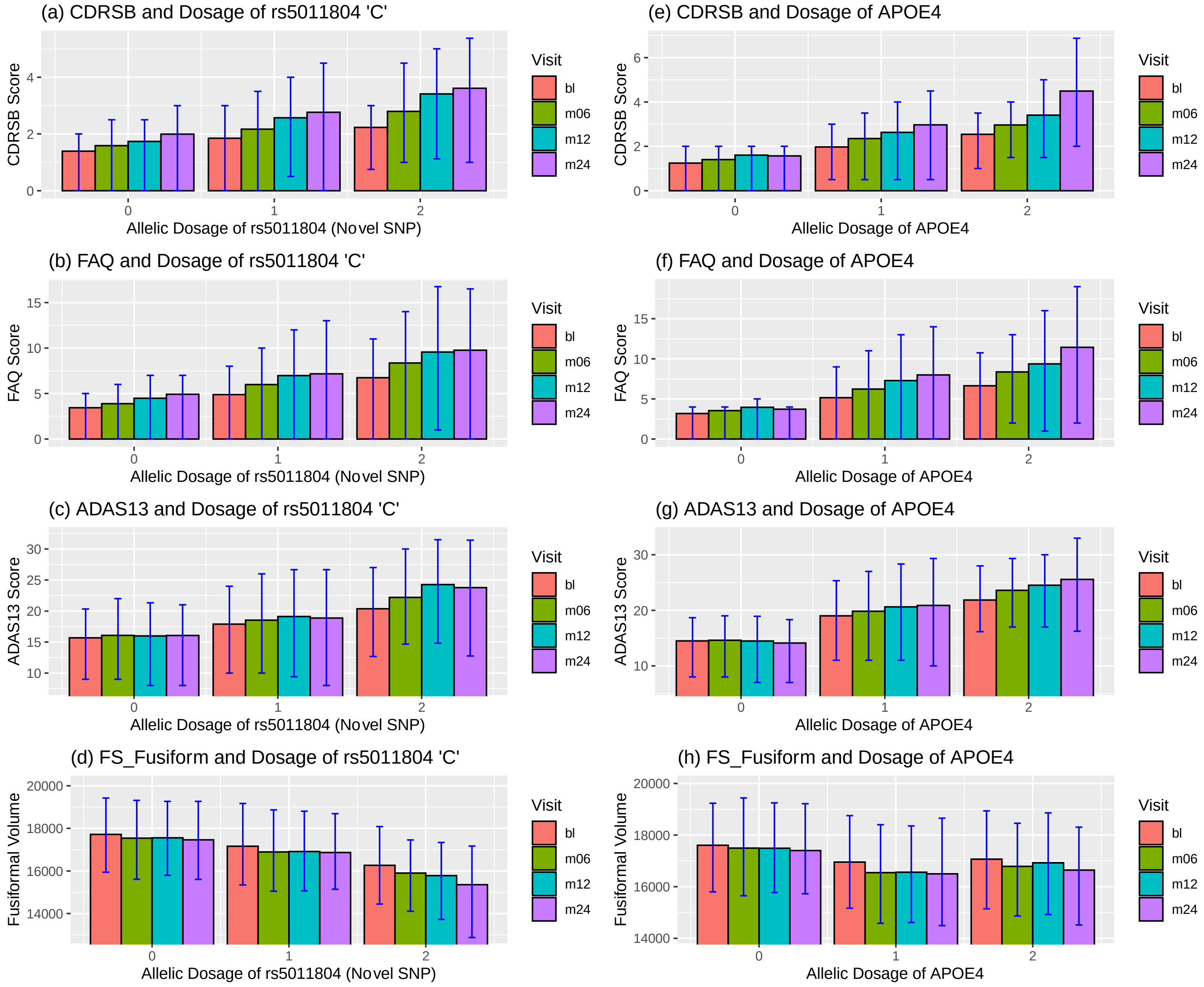

Figure S4: rs5011804-by-diagnosis interaction analysis visualizations. The diagnosis × allelic dosage of the novel SNP rs5011804 was plotted against the median for each of these diagnostic and imaging biomarkers. The error bars shown depict the interquartile range of each measurement.

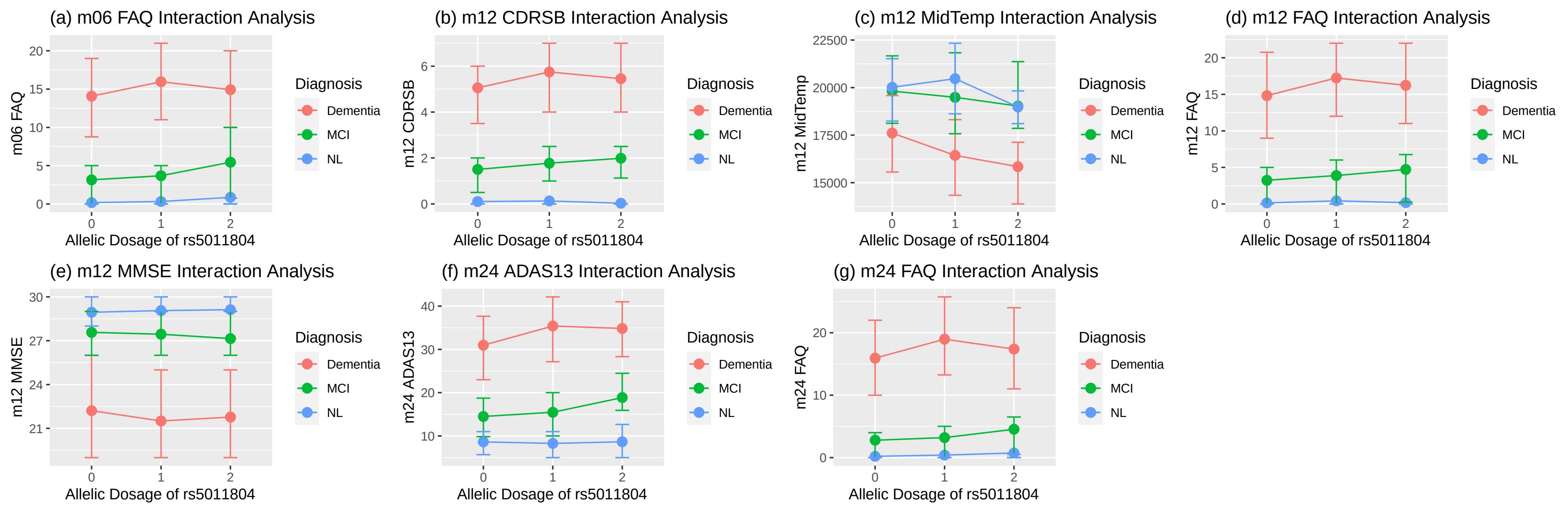

References

[1] PLINK File format reference.
URL http://www.cog-genomics.org/plink/1.9/formats#assoc_linear
